# A rare cause of hematemesis 

**Published:** 2018

**Authors:** Adnan Qureshi, Joanne Cunningham, Ravi Madhotra, Jafer Ali, Kamran Rostami

**Affiliations:** *Department of Colorectal Surgery, Milton Keynes University Hospital, Milton Keynes, UK *

## Question 

An 82-year-old gentleman, who was a nursing home resident, presented with 2 days history of increasing confusion, chest pain and coffee ground vomiting.

PMH includes diabetes mellitus type II, ischemic heart disease, hypertension and dementia. He had no history of fever, abdominal pain, altered bowel habits, weight loss or use of non-steroidal anti-inflammatory drugs. On admission his vital signs were stable. Laboratory markers were unremarkable with Hb% of 14g/dl. Normal CXR, ECG and CT Head. His ABG’s show PH: 7.23, BE: -11, Lactate: 3.1.

**Figure 1:**
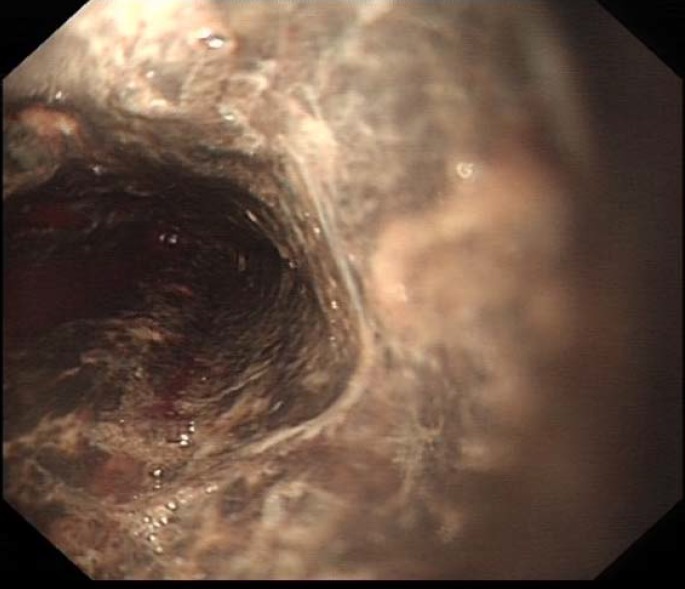
Upper GI Endoscopy shows diffuse, circumferential, discoloured oesophageal mucosa with occasional yellow exudates and signs of friability, loss of light reflex, rigidity and reduced distension of the lumen

**Figure 2:**
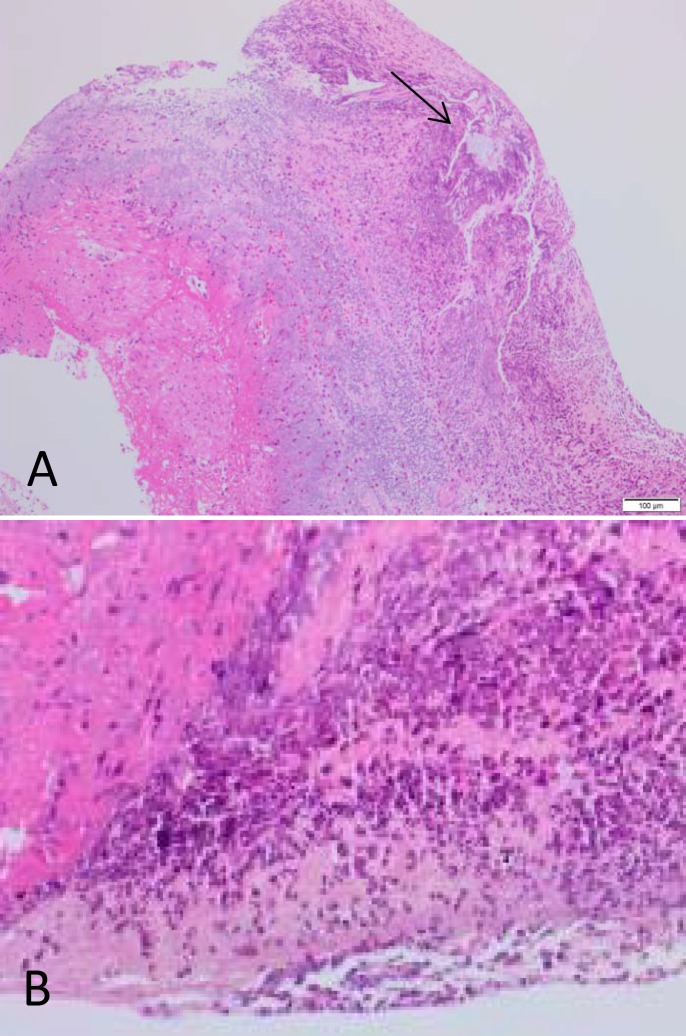
Histology revealed pieces of severely inflamed and necrotic muscularis propria. There are pieces of birefringent crystaline material within the mucosa (Arrow). The squamous epithelium is completely denuded. Fibrinopurulent excudative material and ulcer slough are seen. Special stains show no evidence of fungal organisms (PAS) and gram stain is negative for bacterial colonies. Pearls stain is negative for iron deposition. Immunohistochemistry for CMV and CD20 are negative. CD3 highlights scattered T-cells. Desmin highlights residual muscularis propria destroyed by the ulceration

 After initial resuscitation he had an urgent inpatient upper GI endoscopy. Picture is shown in Figure 1. Histology from the esophagus is shown in Figure 2**.**

**a. What do you think is the diagnosis?**

**b. How would you manage this condition?**

## Black oesophagus or Gurvits syndrome

Acute oesophageal necrosis (Black oesophagus) is a rare syndrome characterised by circumferential black appearance of oesophagus. Commonly involve lower third on oesophagus which has poor blood supply but can involve entire oesophagus. In endoscopy series, the prevalence of acute oesophageal necrosis has ranged from 0.001 to 0.2 percent of cases (1-5). Incidence is higher in males then females.

Etiology can range from ischemia, gastric out let obstruction, antibiotic use, infections (Klebsiella pneumoniae, Candida albicans, CMV & HSV), Diabetic ketoacidosis, and underlying malignancy. These are the patients with multiple co-morbidities, and their prognosis depends on the underlying condition. Mortality rates are variably reported in the literature from 15% to as high as 36% in some cases (6-7). 

Treatment is mainly supportive, addressing the underlying cause, aggressive resuscitation and rehydration. There are controversies about GI feeding, but passage of NG tube should be with caution and preferably undertaken under direct vision during gastroscopy. Early surgical intervention for perforation is advised in suitable candidates. The survivors may develop strictures in (25-30%) which are normally difficult to treat endoscopically (7). 

## Management

He was treated conservatively in the ward with fluid resuscitation, parenteral nutrition and antibiotic and made remarkable recovery. But due to dementia it was a challenge to keep him nourished after stopping parenteral nutrition. Social services, dietitian and occupational therapist helped in planning a safe discharge. 

## Conflict of interests

The authors declare that they have no conflict of interest.
